# Soil-transmitted helminth eggs assessment in wastewater in an urban area in India

**DOI:** 10.2166/wh.2017.147

**Published:** 2017-11-29

**Authors:** Sonia Grego, Viswa Barani, Meghan Hegarty-Craver, Antony Raj, Prasanna Perumal, Adrian B. Berg, Colleen Archer

**Affiliations:** 1 **Sonia Grego**, (corresponding author) **Meghan Hegarty-Craver**, **Adrian B.** Berg RTI International, 3040 E. Cornwallis Road, Research Triangle Park, North Carolina NC-27709, USA; 2 **Viswa Barani** Center for Molecular Medicine and Therapeutics, PSG Institute of Medical Sciences and Research, Peelamedu, Coimbatore 641004, India; 3 **Antony Raj**, **Prasanna Perumal** RTI Global India, 21 Nehru Place, New Delhi 110019, India; 4 **Colleen Archer** Pollution Research Group, University of KwaZulu-Natal, Durban 4000, South Africa

**Keywords:** enumeration, pathogens, prevalence, septage, total solids, viability

## Abstract

Water quality and sanitation are inextricably linked to prevalence and control of soil-transmitted helminth infections, a public health concern in resource-limited settings. India bears a large burden of disease associated with poor sanitation. Transformative onsite sanitation technologies are being developed that feature elimination of pathogens including helminth eggs in wastewater treatment. We are conducting third-party testing of multiple sanitation technology systems in Coimbatore (Tamil Nadu) India. To ensure stringent testing of the pathogen removal ability of sanitation technologies, the presence of helminth eggs in wastewater across the town of Coimbatore was assessed. Wastewater samples from existing test sites as well as desludging trucks servicing residential and non-residential septic tanks, were collected. The AmBic methodology (based on washing, sieving, sedimenting and floating) was used for helminth egg isolation. We tested 29 different source samples and found a 52% prevalence of potentially infective helminth eggs. Identification and enumeration of helminth species is reported against the septage source (private residential vs. shared toilet facility) and total solids content. Trichuris egg counts were higher than those of hookworm and Ascaris from desludging trucks, whereas hookworm egg counts were higher in fresh wastewater samples. Surprisingly, no correlation between soil transmitted helminth eggs and total solids was observed.

## INTRODUCTION

Soil transmitted helminth (STH) infections are a public health concern in resource-limited settings with poor sanitation. While mortality is low, morbidity due to helminth infections is more insidious. Malnutrition, abdominal cramps, diarrhea, and long-term effects like reduced cognitive and intellectual ability, are common problems, particularly in children <5 years for whom consequences are life-long (Bartram & Cairncross 2010).

Helminths reproduce via eggs and sometimes larvae, which are the infective agents transmitted via the fecal-oral pathway (e.g. *Ascaris lumbricoides* and *Trichuris trichiura*) or the skin (e.g. hookworm species and *Strongy-loides stercoralis*). Poor sanitation and drinking water quality are significantly associated with risk of infection (Echazú *et al*. 2015). An effective treatment system must protect people from contact with their excreta and explicitly break the cycle of infection with STHs by ensuring that <1 egg/L is discharged in treated water (WHO 2006). The Reinvent-the-Toilet program (Bill & Melinda Gates Foundation 2011) aims to develop transformative sanitation technologies for onsite wastewater treatment which effectively eliminate or inactivate pathogens, including helminth eggs with a target <0.1 egg/L (IWA 2016).

India bears a large burden of disease, harboring nearly 25% of global cases, with 220.6 million children in need of preventive chemotherapy (Salam & Azam 2017). We are conducting third-party testing of multiple Reinvent-the-Toilet systems in the city of Coimbatore, in the southern Indian state of Tamil Nadu. In order to assess the effective-ness of sanitation technologies in removing or inactivating helminth eggs from treated waste, it is necessary to gather information on prevalence, species and viability of STHs in this area where sanitation system testing sites may be located.

*Ascaris lumbricoides* produces the most resistant eggs, and is also one of the commonest STHs in many developing countries, including Peru, where it was recently found to be most prevalent in domestic wastewater (Yaya-Beas *et al*. 2016). A study on the prevalence and diversity of helminth eggs in pit latrine sludge in Yaounde, Cameroon, found an average of 80 eggs/g dry matter, with *Ascaris* and four other species, including hookworm, being prevalent (Nzouebet *et al*. 2016). In a recent review of 39 studies that reported the prevalence of STH infections from 19 different states in India, *Ascaris lumbricoides* was the most prevalent parasite with >50% prevalence reported from six states, including Tamil Nadu (Salam & Azam 2017). Almost 90% of the studies in Salam & Azam (2017) reported the prevalence of more than one parasite species in the same sample population.

We conducted an exploratory assessment on the presence of helminth eggs in wastewater in the town of Coimbatore by collecting samples from sanitation system test sites as well as randomly sampling from desludging trucks servicing residential and non-residential septic tanks. It was our intent to determine the presence of helminth eggs in these wastewater sources, identify the species found, and determine egg viability or lack thereof.

We adopted the methodology for extracting STH eggs from wastewater and sewage sludge developed by Hawks-worth and Archer (Tronnberg *et al*. 2010) and modified by Archer (Pebsworth *et al*. 2012), known as the AmBic method. This method follows four main principles: (1) washing, (2) sieving, (3) flotation, and (4) sedimentation. The USEPA method (Yanko 1988) is based on the same principles but is much longer and includes an additional extraction step using acetoacetic buffer and ethyl acetate. As ethyl acetate is a solvent which may affect the protective properties of hel-minth egg walls, this step is only used following the AmBic method when it is absolutely necessary and when the assess-ment of viability is not required. Depending on the nature of samples obtained, the AmBic method may be adapted to accommodate them. Thus, very clear water samples may only need to be filtered and centrifuged to sediment a small deposit for microscopic observation, whereas the extraction step may need to be added at the end of the AmBic method if the final deposit is very thick and would otherwise require a very large number of slides to be prepared for microscopy.

This study reports on the presence of helminth eggs in wastewater, assessed in 29 samples from different sources in Coimbatore. We identified and enumerated helminth species eggs, both dead and potentially viable (PV), and examined these results in relation to wastewater source categories (e.g. single family homes versus shared toilets) and a physico-chemical wastewater parameter of total solids content.

## MATERIALS AND METHODS

### Materials

#### Description of source and sampling

A sample size of convenience was used in this exploratory study. Twenty-six fecal sludge samples from randomly selected desludging trucks and three fresh wastewater samples from an apartment complex and a shared toilet were analyzed (*n* = 29 total samples).

#### Fresh wastewater samples

A sample was collected from a sump collecting blackwater from the toilets of an apartment complex. The sump is approximately 1,000 L in capacity and connected to a sewer line. The sump cover was removed and a 5 L container lowered to the bottom using an extension pole and immediately after collection it was poured into a 2 L glass bottle.

Samples were collected in a similar fashion from a shared toilet facility of a construction laborers’ temporary housing complex on PSG Institute of Medical Sciences and Research campus. The shared toilet is used by approximately 450 male construction workers mostly originating from north India. Samples were collected on two separate days from a sump that collects the output of 22 toilet stalls and is connected to the underground drainage system.

#### Desludging truck samples

Samples were collected from 26 desludging trucks discharging their load at the Ukkadam sewage treatment plant (STP) in Coimbatore. The Ukkadam STP is located at GPS (global positioning system) coordinates 10°59ʹ01.1ʺ N76°58ʹ25.7ʺ E.

A stopwatch was used to measure the amount of time that it took the truck to discharge, and grab samples were taken every 30 s (starting with initial discharge) as the truck was emptying using 5 L containers. Three 5 L grab samples, representing the beginning, middle, and end of emptying, were combined to make a 15 L composite sample for each truck. A 2 L sub-sample of the composite sample was reserved for helminth egg screening, 4.5 L was reserved for determining the percentage solids content and the remainder was stored. Information related to the source of the sample (location address, property type, type of sanitation system, inclusion of greywater) was also collected from truck drivers.

Property type included residential (either houses or apartments) sites, commercial sites (e.g. a factory) and public sites (e.g. school, police station). Inclusion of greywater was tracked, since in Coimbatore typically only blackwater from toilets goes into septic tanks, while greywater is drained sep-arately to a soak field. The type of sanitation system describes either septic tanks or storm drains and soak pits.

### Method

#### Helminth egg isolation and enumeration

For helminth eggs assessment, 2 L volumes were obtained for all samples. Some samples had visibly higher solids content than others, consequently sub-samples of 250, 500 or 1,000 mL were tested rather than the full 2 L sample. The 26 samples collected from desludging trucks were partially processed and stored for analysis to be conducted at a later time; the fresh samples collected from the shared toilet and apartment complex were analyzed immediately.

**Figure 1:**
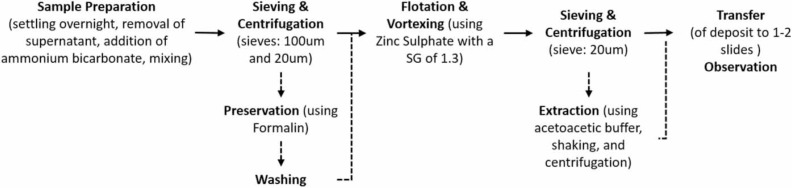
Summary of helminth egg isolation methodology.

The preparation was as follows (see Figure 1): sample volumes were measured into plastic beakers and allowed to settle overnight. The supernatant water was carefully suc-tioned off, an amount of ammonium bicarbonate (AmBic; 119 g/L) equal to the volume of the sediment in the beaker was added to the deposit and mixed on a magnetic stirrer with the aid of a bar magnet for 10-15 minutes. The mixture was then filtered through a 100 pm, 200 mm diameter stainless steel pan sieve onto a 20 pm sieve fitted beneath it. The sample was washed well with distilled water under pressure from an adapted fertilizer pump (Jawan Brand, 16 L capacity model) to allow all helminth eggs to pass through and be retained in the filtrand on the 20 pm sieve. The sieves were then separated, all the filtrand pipetted into either 50 mL or 15 mL Falcon tubes (depend-ing on the amount of sediment), and the tubes centrifuged at 1,389 g for 15 minutes. The supernatant water was removed and an amount of 2.5% formalin added that was equal to the sediment volume in the test tube. The samples were then stored at 4°C for about one month.

In order to continue processing the stored samples, they had to be washed twice in distilled water to remove the formalin and all supernatant fluid was discarded. The deposit remaining in a 15 ml tube was required to be ≤1mL and in a50 mLtube ≤5 mL. So, when this was not the case, the deposit was shared among two or more tubes to accommodate this. The flotation step was then performed using zinc sulfate (ZnSO_4_) at a specific gravity (SG) of 1.3 to allow for all STH eggs to float: each tube was filledtothe 14 mL/45 mL mark while vortexing them with an applicator stick in the tube to act as a stirring rod. Next they were centrifuged at 617 g to float the eggs. The entire supernatant fluid was poured over a20 nm pore, 100 mm diameter stainless steel pan sieve and washed well with distilled water from the fertilizer pump. All the filtrand was then returned to one or more test tubes and centrifuged at 1,389 g to obtain a deposit and the supernatant fluid was discarded. If the deposit was in a quantity smallenoughtoeasilytransfertooneortwomicroscope slides, then this was the final step of sample preparation.

If the deposits were a larger quantity, then the extraction step was added. This step involved the addition of 7 mL acetoacetic buffer (15 g sodium acetate tri-hydrate plus 3.6 mL glacial acetic acid made up to 1 L with distilled water) and 3 mL of ethyl acetate, vigorous shaking for one minute, centrifugation at 1,389 g for 15 minutes, removal of all supernatant fluid, with the resulting pellet ready for microscopic examination. We included this step on 15 of the 26 truck samples and both shared toilet samples.

All deposits were examined using a compound microscope (LX400, Labo America, Fremont, USA) at 100× and 400× magnification, helminths identified to genus or species level and enumerated accordingly.

The average STH egg concentration in wastewater was defined as the number of eggs/L for positive samples only (i.e. samples with no eggs were excluded from the calculation):

$$\text{Concentration}\left(\frac{\text{eggs}}{\text{L}}\right)\text{=}\frac{\text{Number of eggs}}{\text{Volume (L) of positive sample}}$$ (1)

Percentage was defined as the number of positive samples divided by the total number of collected samples,

multiplied by 100:

$$\text{Prevalence}\left(\text{\%}\right)\text{=}\frac{\text{Number of positive samples}}{\text{Number of Samples}}\text{*100\%}$$ (2)

#### Total solids determination

The 4.5 L sample was initially solar dried in plastic containers to remove the majority of the water. Then, the samples were carefully transferred to dry Petri dishes that had been previously weighed. The weight of the sample and the Petri dish were recorded, and the samples placed overnight in a temperature-controlled oven that was maintained at 50-65°C. The samples were removed, allowed to cool for 15 minutes and the mass re-measured. The samples were dried for another 4 hours at 50-65^°^C, cooled and the mass re-measured. The 4 h drying process was repeated until the dry mass before and after drying varied by <4%. The percen-tage solids content was calculated as:

$$\text{\% Solids =}\frac{\text{Dry mass (g) - mass of petri dish (g)}}{\text{Initial sample volume (mL)}}\text{*100\%}$$ (3)

## RESULTS

### Identification of STH eggs

Human helminth eggs found in the desludging truck samples were those of the three commonest STH species, namely *Ascaris, Trichuris* and hookworm, as well as two tapeworms, *Hymenolepis nana* and *H. diminuta*. Other eggs present were *Aspiculuris* sp., *Heterakis spumosa* and *Trichosomoides crassicauda* (common rat parasites) and *Calodium hepaticum*, a liver parasite of mammals very common among rats (old name, *Capillaria hepatica*). Egg counts of PV eggs of individual species ranged from <1 to 56 eggs/L.

At the shared toilet, two fresh sewage samples were col-lected on different days: the first had 100 *Ascaris* sp., 68 *Trichuris* sp., and 49,792 hookworm sp. eggs/L; and the second, 16 *Ascaris* sp., 8 *Trichuris* sp., 556 hookworm sp., 4 *H. nana* and 2 *Trichostrongylus*-like eggs/L. One 2 L sample was taken from the apartment sump. A 500 mL sub-sample was processed and examined and 2 dead *Ascaris* eggs plus 52 hookworm sp. eggs/L were found.

All egg counts were converted to eggs per liter (Table 1, Supplementary material, available with the online version of this paper). Eggs that looked in good condition were cate-gorized as PV and reported separately from those that were obviously dead (D). Included in the dead eggs were infertile eggs (I) as they cannot develop further.

**Figure 2:**
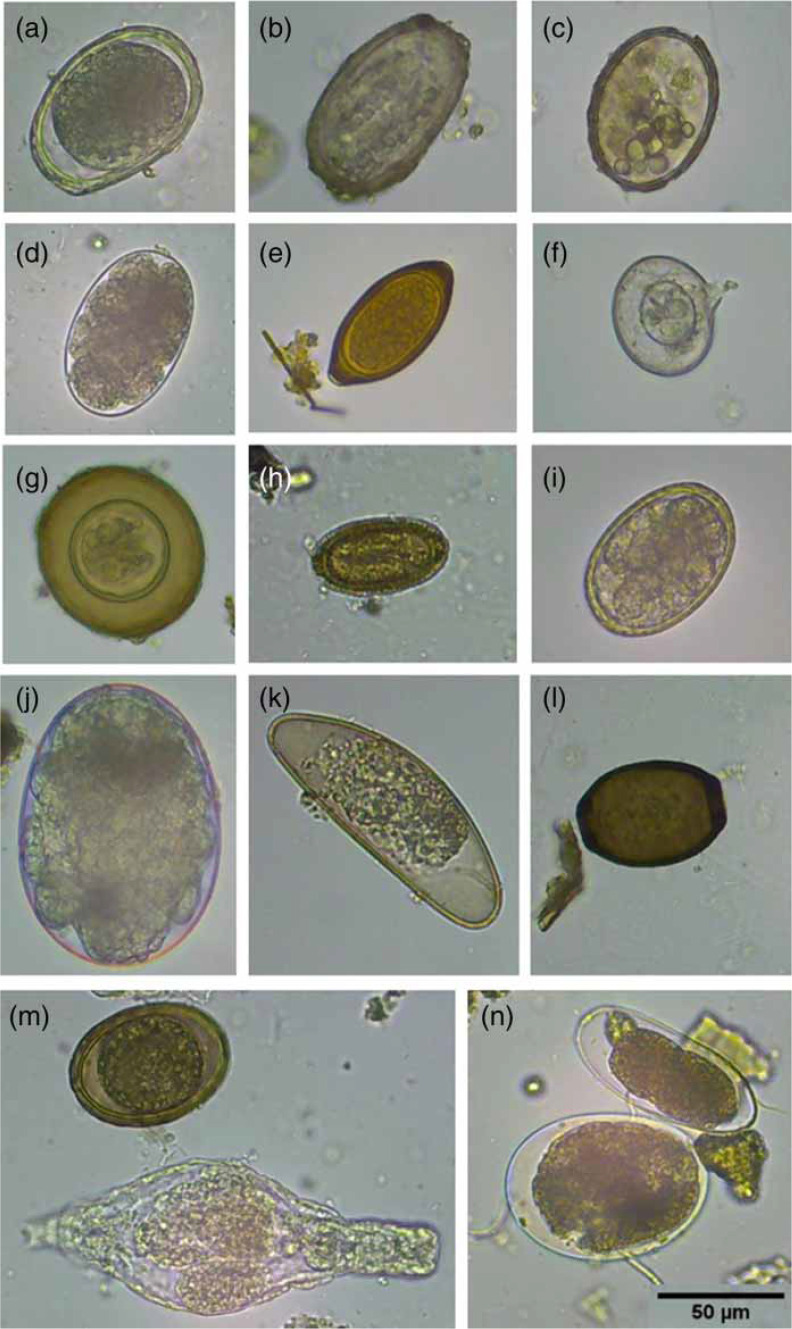
(a) Undeveloped Ascaris sp. egg (PV); (b) Ascaris sp. egg containing necrotic larva; (c) dead (globular) Ascaris sp. egg; (d) developing hookworm sp. Egg (PV); (e) undeveloped Trichuris sp. egg (PV); (f) dead (broken wall) Hymenolepis nana egg; (g): (h). diminuta egg (PV); (i) developing Heterakis spumosa egg (PV); (j) developing mite egg; (k) Aspiculuris sp. egg (bad condition); (l) dead Trichosomoides crassicauda egg; (m) undeveloped (PV) Ascaris (top) and rotifer (bottom); (n) unknown hookworm-like, but too long, egg (top) and undeveloped mite egg (bottom). Scale bar (50 μm) for all photographs in bottom righthand corner.

Photomicrographs of some of the eggs which were found in the samples tested are shown in Figure 2. All nematode (roundworm) eggs are laid in the undeveloped, single cell stage and develop to 2, 4, 8, >16 cells until a L1 larva forms and moults to the infective L2 stage. Eggs at any of these stages were classed as 'PV'. If an egg had a larva inside that looked nice and plump and alive, but was immotile, we also described it as 'PV', and if it was motile, it was viable. If the larva looked thin and shrivelled, it was referred to as necrotic and fell under the category, 'dead'. In the undeveloped or developing stages, eggs with a cracked or broken shell, or a dented and folded-in outer shell, both with nothing visible inside the egg, or eggs with globules inside were considered dead. Infertile eggs are elongated and narrower than normal eggs and cannot develop as they have not been fertilized. As female nematodes lay very large numbers of eggs per day, some simply do not get fertilized (a natural phenomenon in most animals). On occasion, there are no male worms present in an infection and then in this case all eggs produced by the infected person are infertile and of no consequence as they pose no infection risk for others.

### Helminth egg prevalence and intensity in fecal sludge samples

Twenty-six fecal sludge samples from desludging trucks and three fresh fecal sludge samples from an apartment complex and a shared toilet were analyzed (*n* = 29 in total). Both dead and PV helminth eggs were counted. *Ascaris*, *Trichuris*, and hookworm sp. eggs were distinguished separately from 'other potentially pathogenic' (other PP) spp. eggs which included *Hymenolepis diminuta, H. nana*, and *Capillaria hepatica*. 'Other non-pathogenic' (other NP) spp. eggs included *Trichosomoides crassicauda, Aspiculuris* sp., and *Heterakis spumosa*, which are infective for rats but not humans.

**Figure 3:**
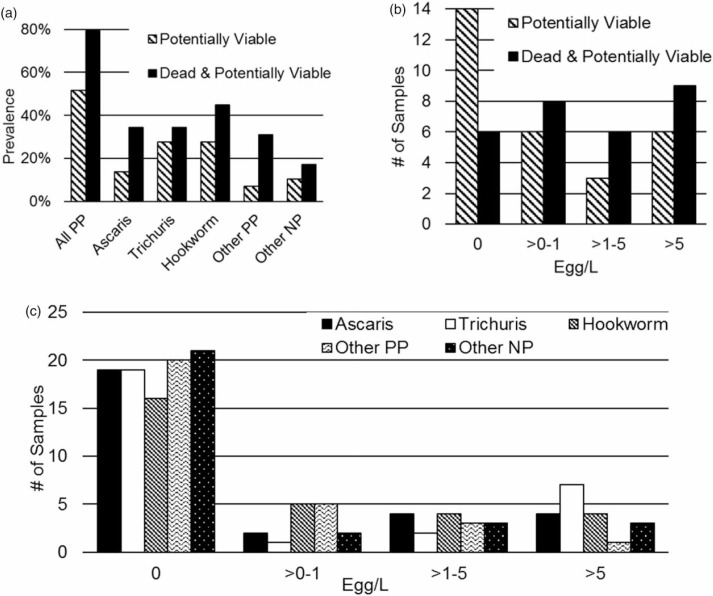
(a) Prevalence of helminth spp. eggs by individual species (*n* = 29). (b) Distribution of total counts of helminth spp. eggs (*n* = 29). (c) Distribution of counts of helminth spp. Eggs by individual species (*n* = 29). PP: potentially pathogenic; NP: non-pathogenic.

Prevalence was computed for both dead and PV pathogenic eggs. Figure 3(a) reports the prevalence for all the species in the first bar data and break down by species. A prevalence of 52% was recorded for PP viable eggs and 79% when both dead and PV pathogenic eggs were included (Figure 3(a)). When examining prevalence by species, and when only PV pathogenic spp. eggs were considered, *Trichuris* and hookworm (28% each) were highest, followed by *Ascaris* (14%). There was a similar trend when all eggs were included (Figure 3(a)).

For illustrative purposes, samples with egg counts were equally distributed into three categories by egg concentration (0-1, 1-5, and >5 eggs/L) (Figure 3(b)). Fractional numbers of eggs/L are interpreted as finding a single egg in a sample volume > 1 L and 8 out of the 29 samples exhibited this low level of contamination. Figure 3(b) indicates that only 9 out of 29 samples had more than 5 eggs/L, indicating high level of dilution. The distribution by species versus count is shown in Figure 3(c), where *Trichuris* was the most prevalent species with egg concentrations > 5 eggs/L.

**Figure 4:**
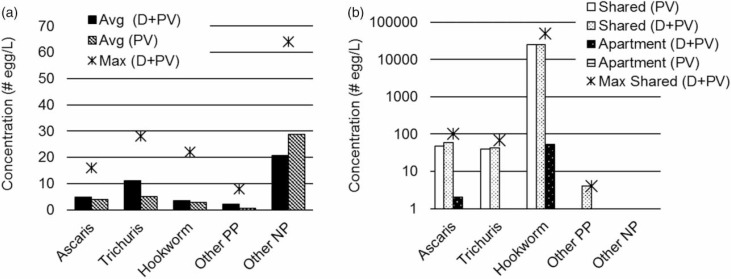
(a) Average concentration of helminth spp. eggs by individual species from desludging trucks (*n* = 26). (b) Average intensity of helminth spp. eggs by individual species collected from sumps (*n* = 3). D = dead; PV = potentially viable.

The average egg concentration recorded in this study for desludging trucks was <10 eggs/L for all species, with the exception of a few samples with high counts of rat parasite eggs that raised the average of the NP eggs shown in Figure 4(a). Samples from fresh wastewater exhibited much higher concentrations of eggs (Figure 4(b)), a factor of 10 and 100 times higher than for the desludging truck samples. Figure 4(b) is therefore plotted on a log scale and it also includes the maximum recorded concentration value for each species.

### Effect of the septage source

We further examined the egg prevalence in desludging trucks only. A prevalence of 77% (*n* = 26) across these samples was found when both dead and PV pathogenic eggs were considered (the prevalence was 50% when only PV pathogenic eggs were counted). The average concentration of pathogenic helminth spp. eggs was low across these 26 samples (dead and PV: 8.7 eggs/L; only PV: 4.3 eggs/L).

For analysis of results, sample sources were divided into five different categories: house, apartment, shared, drainage, and other. Residential sources included septic tanks, either from houses or apartments. The 'shared' category included public toilets and institutional settings where multiple users accessed the same facility (i.e. a school and a police station); the 'drainage' category included storm drains and soak pits and was expected to have a high ratio of greywater to blackwater; and the 'other' category included sources from commercial and industrial settings.

**Figure 5:**
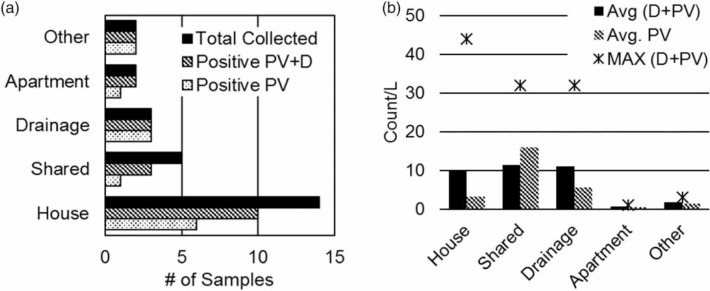
(a) Prevalence of helminth spp. eggs from desludging trucks by septage sources (*n* = 26). (b) Average concentration (excluding 0 counts) of helminth spp. eggs from desludging trucks by septage source (*n* = 26). PV = potentially viable; D = dead.

The majority of the 26 samples (*n* = 14) came from houses, followed by shared toilet facilities (*n* = 5). Positive samples were found across all sources, although many samples from houses and shared toilet facilities tested negative for helminth spp. eggs (Figure 5(a)). The average concentration of PV helminth spp. eggs was highest in the shared toilet and drainage samples (Figure 5(b)); however, average concentration counts were not high (<15egg/L) and the maximum recorded concentration (44 egg/L) was from a house.

### Effect of septage solids content

Total solids content was evaluated for the samples collected from the desludging trucks and was found to be highly variable, ranging from <0.1% to 7%.

For graphical illustration of results, the samples were divided into three categories according to their solids content to give approximately equal numbers of samples across each: ‘low’ if the solids content was <1% (*n* = 10), ‘medium’ if the solids content was between 1 and 3% (*n* = 7), and ‘high’ if the solids content was >3% (*n* = 9).

**Figure 6:**
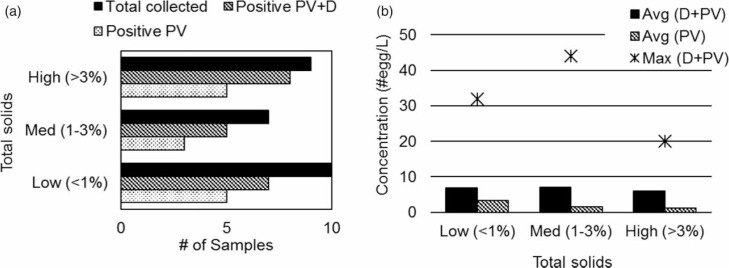
(a) Prevalence of helminth spp. eggs from septage with different solids content (*n* = 26). (b) Average concentration and maximum counts/L in septage by solids content (*n* = 26). PV = potentially viable; D = dead.

Helminth egg prevalence among each category of total solids content was similar to the overall prevalence for the desludging truck samples (Figure 6(a)), indicating that prevalence was unrelated to solids content.

The average concentration was low (<10 eggs/L) across all categories, and also did not depend on solids content (Figure 6(b)). The highest concentration recorded was 44 eggs/L. This sample had a solids content of 1.2% and was from a single-family house.

## DISCUSSION

This study explored the prevalence and concentration of STHs in wastewater samples in an Indian urban area, the city of Coimbatore, one of the sites where Reinvent the Toilet Technologies are to be tested. For assessing the ability of sanitation technologies to achieve biologically safe treated waste, it is important to additionally assess the viability of the eggs. We determined viability by using rigorous criteria including morphological analysis and presence of visible larvae as observed by an experienced microscopist.

We recorded a 52% prevalence of samples with PV and therefore infective helminth eggs in 29 samples from different sources. A higher prevalence of 72% was obtained if we included both dead and PV eggs.

The concentration of helminth eggs among positive samples was relatively low, with many samples having only a few eggs/L, and average concentration in the order of 10 eggs/L. This STH egg concentration in wastewater points to a health risk for infection if the waste is not properly managed. It is, however, lower than the loads measured in other Indian wastewater studies ranging from 50 to 100 eggs/L (Yaya-Beas *et al*. 2016) and orders of magnitude lower than those measured in pit latrine sludge in Africa of 30,000 eggs/L (Nzouebet *et al*. 2016). This would indicate that a fraction of individuals are respon-sible for pathogens in environmental wastewaters. Our result is consistent with data previously reported for the state of Tamil Nadu (Salam & Azam 2017) which indicated a 50% prevalence of infection.

Furthermore, a high load of helminth eggs was found in the shared toilet of construction laborers, not surprisingly due to the low socio-economic status and work environment of these individuals.

Nine different species of helminth eggs were identified in this study. We note that the isolation process adopted a solution with specific gravity SG = 1.3 for the flotation step, a SG value well suited to recover all species of helminth eggs with relative densities ranging between 1.06 and 1.24 g/cm^3^ (da Rocha *et al*. 2016).

In East Africa, *Ascaris, Trichuris* and hookworm sp. were reported as equally prevalent (Brooker *et al*. 2009). We, how-ever, found that Trichuris was the commonest helminth as defined by the number of samples with > 5eggs/L (Figure 3(c)), and highest average count by species (Figure 4(a)). Hookworm sp. eggs were present in the highest concentration (up to 49,000 eggs/L) in fresh wastewater samples from the shared toilet (Figure 4(b)). Although Ascaris eggs were also present, they were not the main species. It is possible that hookworm is the most prevalent species in infected individuals but not in samples obtained from septic tanks. We hypothesize that hookworm sp. eggs are (1) not as hardy as those of *Ascaris* or *Trichuris* (Obeng & Wright 1987) and thus do not survive the longer time in septic tanks compared with fresh wastewater, or (2) they develop much faster than *Ascaris* and *Trichuris* (as frequently observed in school surveys conducted in multiple African countries from 2010 to 2015 by Colleen Archer [last author of the present paper]), hatch to rhabditiform and then filari-form larvae, and die due to the inability to infect a new host (via the skin) while being contained in a septic tank.

This could also account for the absence of *Strongyloides stercoralis* larvae in any of the samples of this study, although it is highly likely that this helminth is present in the same sort of geographical locations as hookworm spp. (Beaver *et al*. 1984).

The prevalence of rat parasites, *Trichosomoides crassicauda, Aspiculuris* sp., and *Heterakis spumosa* eggs, plus Capillaria hepatica (34%; *n* = 9/26) was comparable to human pathogenic STH eggs in samples collected from the desludging trucks, while these species were not observed in fresh samples. This may indicate that rat access to, or rat excreta contamination of, septic tanks is an issue. The first three species are infective to rats, but not humans, whereas although *C. hepatica* is mostly transmitted by *Rattus* spp. within cities, it is infective to a large number of mammals including humans. This observation therefore points to a health risk.

We analyzed the effect of the wastewater source on the prevalence of helminth eggs to evaluate whether shared facilities may be more likely to be positive for STHs than individual houses; however no effect was recorded and positive samples and counts were similar.

Due to their larger size, helminth species eggs are known to adhere to solid surfaces and settle more easily in sludge than bacteria and viruses (Shamma & Al-Adawi 2002). STH eggs are therefore expected to be in higher concentrations in samples with higher total solids derived from settled sludge. We measured the total solids in desludging truck samples and found a large variability in solids content, consistent with observation by others in desludging trucks from a different city in Tamil Nadu (Krithika *et al*. 2017). This variability is attributed to many factors including the suction power of the vacuum pump on the truck, the collection of supernatant from the septic tank rather than the sludge, and different levels of sludge accumulation at the source (as determined by different cleaning frequencies). The prevalence and concentration values of STH eggs do not vary significantly across different solids contents (Figure 6(a) and 6(b)).

The lack of correlation between helminth egg presence and total solids is surprising. It has been observed that solids interfere with STH recovery (Amoah *et al*. 2017); how-ever we believe this was not the case in this study for two reasons: (1) the total solids values below 5% - the range in our study - are generally not considered problematic (Amoah *et al*. 2017); and (2) the AmBic method is well-suited to isolate eggs from soil particles and is therefore effective for samples with high solids content. We therefore believe that the reported data reflect the actual content of the samples and that the health risk posed by fecal sludge from septic tanks in this area is independent of the solids content of the sample and is therefore also independent of whether the trucks collect from the sedimented sludge or the supernatant of the septic tank.

In conclusion we report that STH eggs are common in wastewater in the urban environment selected as a test site for Reinvent-the-Toilet technologies in southern India. The described approach to isolation, enumeration and viability assessment of STH eggs is well suited to determine the effectiveness of waste treatment by transformative sanitation technologies that will be a key tool towards eradication of these infections.
