# Supplementary material for: Soil-transmitted helminth eggs assessment in wastewater in an urban area in India
Source: J Water Health. 2017 Nov 29;16(1):34–43. doi: 10.2166/wh.2017.147 (PMC7734373; doi:10.2166/wh.2017.147)

**Supplementary Material**

Table 1 reports the data of helminth egg identification by sample that were used to generate the graphs of the result sections in Figures 3, 4, 5 and 6. The samples are labelled as T1 through T26 for the trucks and F1 through F3 for the fresh wastewater samples. For each sample, its source, the egg counts by species and viability status and total solids are reported.

Table 1: Detailed results of microscopic examination for helminth eggs (count in eggs/L; D = dead; PV = potentially viable)


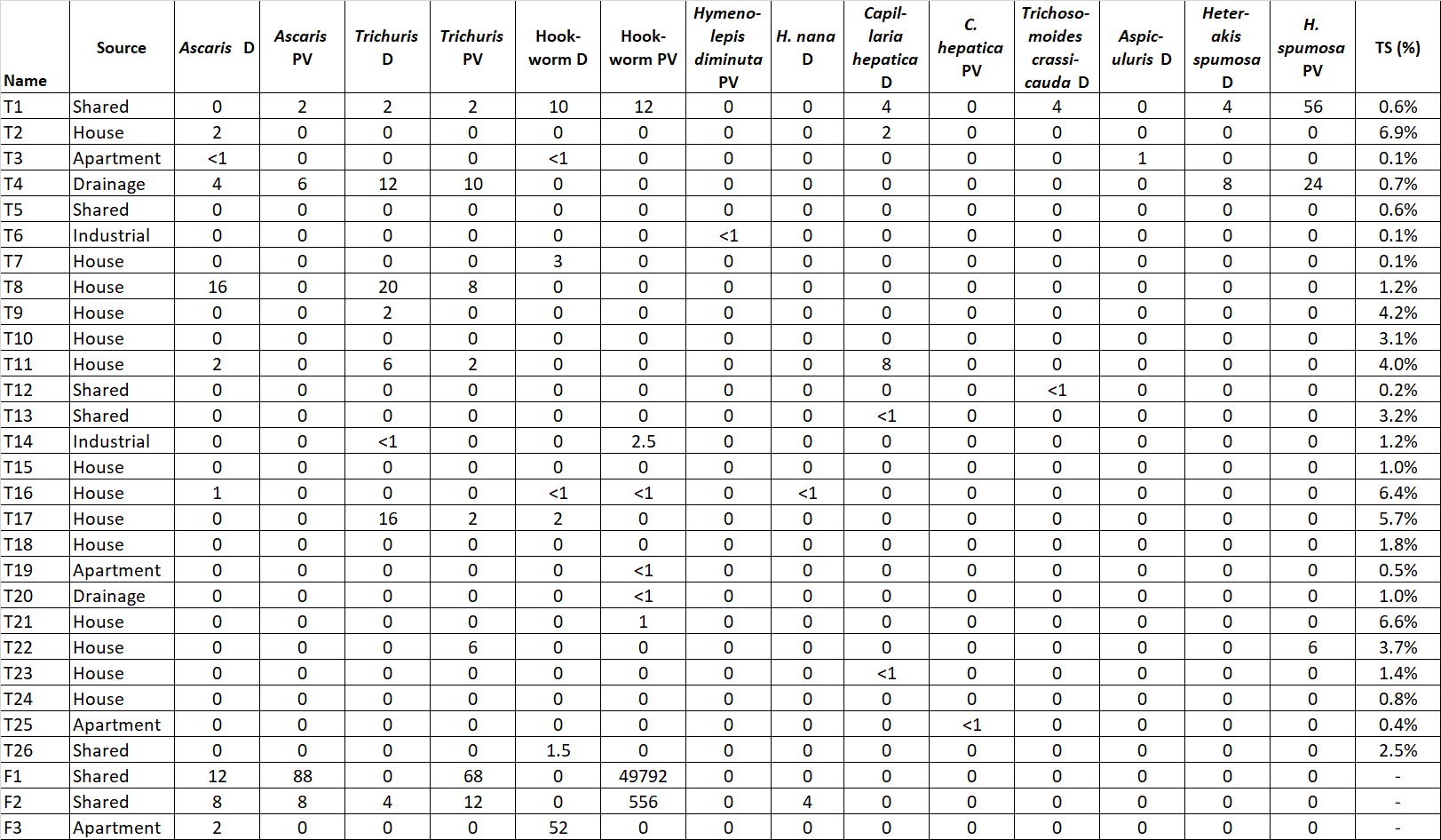

Supplement: Supplementary file 1 [file JWH-16-01-034-s001.docx]
